# Autopolymerizing acrylic repair resin containing low concentration of dimethylaminohexadecyl methacrylate to combat saliva-derived bacteria

**DOI:** 10.1007/s10856-022-06670-7

**Published:** 2022-05-31

**Authors:** Wen Zhou, Hongyan Zhao, Zhen Li, Xiaojing Huang

**Affiliations:** Fujian Key Laboratory of Oral Diseases & Fujian Provincial Engineering Research Center of Oral Biomaterial & Stomatological Key lab of Fujian College and University, School and Hospital of Stomatology, Fujian Medical University, Fuzhou, China

## Abstract

Biofilm accumulation on the polymethyl methacrylate (PMMA) restorations negatively affect the prognosis of the provisional restorations or the following treatment. This study developed a novel antibacterial PMMA resin containing low concentration of dimethylaminohexadecyl methacrylate (DMAHDM). Four resins were tested: (1) PMMA resin (Control), (2) 1.25% DMAHDM, (3) 2.5% DMAHDM, (4) 5% DMAHDM. Adding 1.25% DMAHDM into the PMMA resin did not influence the mechanical properties, degree of conversion, monomer releasing, and color stability of the specimens (*p* > 0.05). The incorporation of DMAHDM into PMMA resin could greatly prevent saliva-derived biofilms adhesion compared with the control group (*p* < 0.05). The metabolism level of saliva-derived biofilms on the 1.25%, 2.5%, and 5% DMAHDM resins were reduced by 20%, 54%, and 62%, respectively. And the mechanism of DMAHDM disturbing the integrity of bacterial cell walls was confirmed by flow cytometric analysis. Adding 1.25% and 2.5% DMAHDM did not compromise cytocompatibility of the modified resin (*p* > 0.05). Therefore, novel PMMA resin containing low concentration DMAHDM is promising as a future antimicrobial provisional restoration material for preventing microbial-induced complications in clinical settings.

**Figure:**
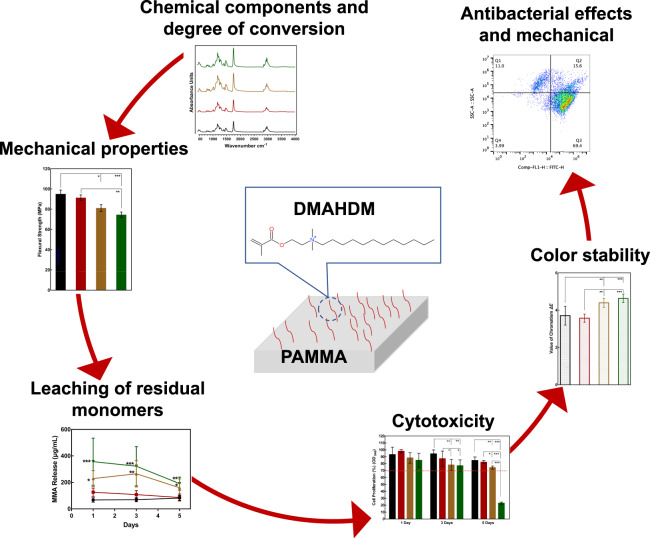
Graphical abstract

Graphical abstract

## Introduction

Provisional restoration is an essential part of fixed prosthodontics, dental implant treatments, and other dental treatments [1, 2]. They are important in determining the prognosis of these treatments [1, 2]. Given the increasing aesthetically driven demand to reduce the edentulous period, implant-supported immediate provisionalization has been suggested as a viable option [3]. Temporary restorations help to shape the soft tissue profile around the prepared tooth, pontics and implant abutments to achieve healthy soft tissue development and meet aesthetic demands [2, 4, 5]. In other circumstances, the provisional restorations protect the pulpal tissue against thermal, mechanical, and physical irritation and bacterial contamination [2].

However, compared with the final restorations, the provisionals have higher surface roughness and less marginal adaptation, making them more vulnerable to biofilm attachment. Biofilm accumulation on the provisional restorations causes gingival inflammation, denture stomatitis, and secondary caries [2]. Autopolymerizing acrylic repair resin, the polymethyl methacrylate (PMMA), is still one of the most common denture repair materials, allowing the denture repairs to be easily completed at room temperature in a short period without the use of any additional equipment [6, 7]. However, the PMMA has been found to lack antibacterial effects [1]. A possible solution to this problem is to engineer materials with antibacterial properties [1, 8]. Previous researchers have explored attempts to produce resin composite with antibacterial properties by incorporation of antimicrobial drugs, such as chlorhexidine, silver, graphene oxide, and platinum nanoparticles [9–11]. However, the addition of these substances sometimes negatively influences the mechanical and aesthetic properties of PMMA [1, 12]. And these antimicrobial drugs would be released, without the potential for long-lasting microbial antibacterial effects [1, 12].

Quaternary ammonium methacrylates can copolymerize to dental methacrylate monomers in the resins, thus exhibiting long-term antibacterial effectiveness [13–20]. A new antibacterial monomer dimethylaminohexadecyl methacrylate (DMAHDM) was incorporated into dental resins with potent and broad-spectrum antibacterial effects activity [21, 22]. Recently, DMAHDM and chlorhexidine were used to modify PMMA resins, but the high concentration of DMAHDM (mass fraction of 5%) hindered the mechanical and aesthetic properties of the resin [23]. Nowadays, using antibacterial agents in low concentration is suggested to limit the interference with the materials’ properties and allergic simulation to the host [24].

Therefore, the objectives of this study were to develop an antibacterial PMMA resin with low concentration of DMAHDM; investigate its physicochemical and aesthetic properties and biocompatibilities; and determine its antibacterial effects and mechanism against saliva-derived biofilm for the first time. It was hypothesized that: (1) the incorporation of DMAHDM in low concentration would not influence the physicochemical and aesthetic properties and biocompatibilities of the modified resin when compared with the Control group; (2) the new antibacterial PMMA resin would effectively inhibit the saliva-derived biofilm.

## Materials and methods

### Fabrication of samples

DMAHDM (purity > 95%) was synthesized using a modified Menschutkin reaction where by a tertiary amine (2-(dimethylamino) ethyl methacrylate) was reacted with 1-bromohexadecane (organohalide) [25]. For the fabrication of PMMA acrylic resin with DMAHDM, DMAHDM was dissolved in the liquid monomer in proportions equivalent to 1.25%, 2.5%, and 5% of the weight of the polymer. Then the self-cured acrylic resin was prepared according to the manufacturer’s recommendations (#1, New Century, China). Thereafter, as recommended in previous studies, post-polymerization heat treatment was performed to improve the degree of conversion (DC) while reducing the leaching residual monomers of methyl methacrylate (MMA) [26]. The samples were immersed in water at 60 °C for 1 h [7]. Therefore, the following four acrylic resins were tested:

1. PMMA acrylic resin (referred to as Control);
2. PMMA acrylic resin with 1.25% DMAHDM (referred to as 1.25% DMAHDM);
3. PMMA acrylic resin with 2.5% DMAHDM (referred to as 2.5% DMAHDM);
4. PMMA acrylic resin with 5% DMAHDM (referred to as 5% DMAHDM).

### Chemical components and DC assessment

Resin disks were prepared using molds with a diameter of 9 mm and thickness of 1 mm. Each open side of the disks was covered with Mylar strips and self-cured for 15 min [23]. After that, post-polymerization heat-treatment was performed in 200 mL of deionized water (DDW) and stirred with a magnetic bar at 60 °C for 1 h [7]. Then the disks were sterilized in ethylene oxide (SQ-H40, Sanqiang, China) and degassed for 7 days.

Specimens were crushed with a mechanical mill (BSH-C2, Life Real, China). Resin powder of 1 g for each group was obtained for the test. The chemical components and DC were determined with Fourier transform near-infrared spectroscopy. Scans were completed over a spectral range of 400–4000 cm^−1^.

The absorbance peaks at 1637 cm^−1^ and 2952 cm^−1^ correspond to aliphatic (C=C) double bonds and aliphatic (C–C) bonds. Absorbance peak intensity values of C=C and C–C in unpolymerized and polymerized specimens were proportioned, and DC values were recorded as percentages (%) [7].

### Mechanical testing

Samples were made by a rectangular mold of 2 × 2 × 25 mm [27, 28]. Then post-polymerization heat-treatment was conducted. The specimen was fractured in three-point flexure with a 10 mm span at a crosshead-speed of 1 mm/min on a computer-controlled universal testing machine (5500 R, MTS, USA) [29]. Flexural strength was calculated as: S = 3P_max_/L(2bh^2^), where P_max_ is the fracture load, L is span, b is specimen width and h is thickness. Elastic modulus was calculated as: E = (P/d)(L^3^/[4bh^3^]), where load P divided by displacement d is the slope in the linear elastic region. Six specimens were tested for each group (*n* = 6).

### Determination of leaching residual MMA concentration

**Fig. 1:**
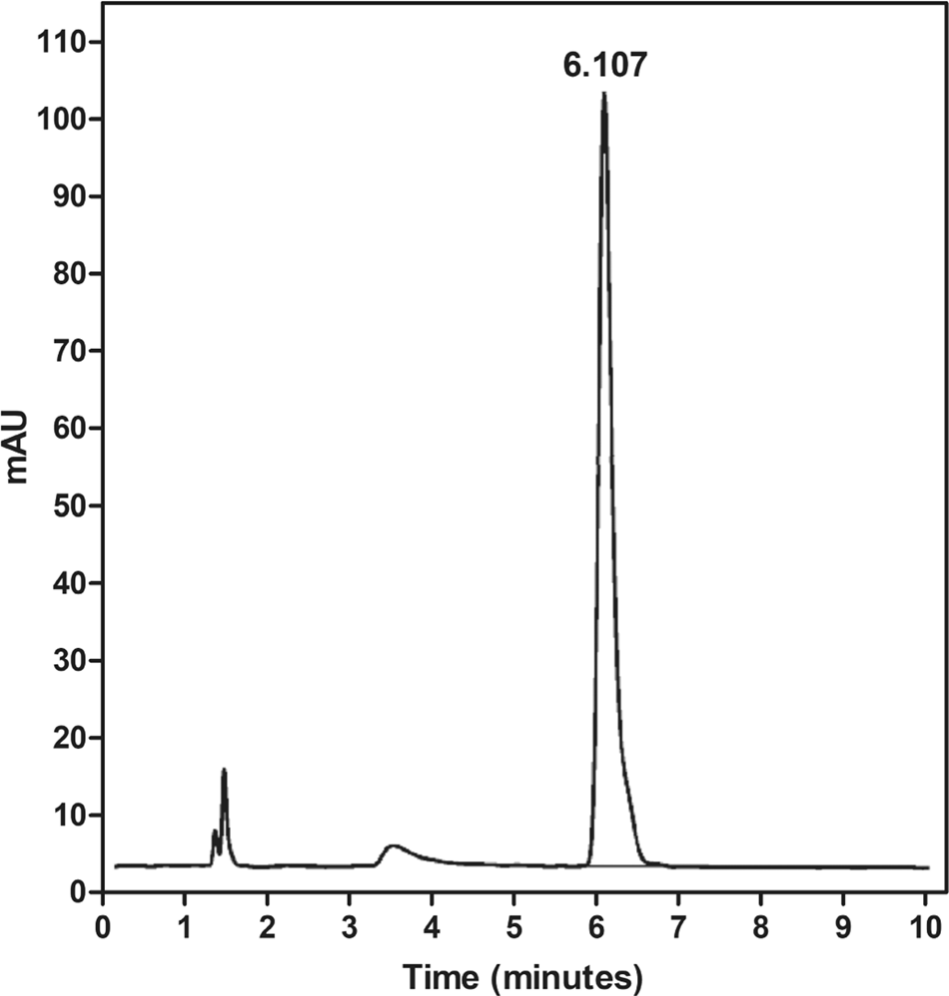
HPLC chromatogram of methyl methacrylate (MMA) and peak at 6.017 min of retention time

Eluates were prepared by placing each resin disk into 3.54 mL of methanol [7]. Elution of the specimens was conducted for 1, 3, and 5 days at 37 °C (*n* = 6). After each time point, the eluates were collected and the specimens were transferred into new tubes with fresh methanol. Residual MMA concentration in the eluates was determined via high performance liquid chromatography (HPLC) (G7104C, Agilent, USA). Known serial concentrations of 0, 2, 4, 8, 16 and 32 μg/mL of MMA dissolved in methanol were analyzed, and a calibration curve was obtained using chromatographic MMA peak at 6.017 min of retention time (Fig. 1).

### Cytotoxicity determinition

Eluates were prepared using complete cell culture medium without serum at the same ratio of surface area of the discs to the volume as in Section *Determination of leaching residual MMA concentration*. Elution of the specimens was conducted for 1, 3, and 5 days at 37 °C under an atmosphere of 5% CO_2_, 95% air (*n* = 6). Complete cell culture media without specimens were also incubated to serve as negative controls. Eluates were filtered for sterilization and 10% fetal bovine serum (Gibco, USA) was added. Then the eluates were stored at −20 °C.

Primary human gingival fibroblasts (HGFs) were obtained from Fujian Key Laboratory of Oral Diseases. 5000 cells/well were seeded in 96-well plates. The cells were cultured for 24 h. Then the medium was replaced with 100 μL eluates, and incubation for another 24 h was conducted. Thereafter, 100 μL of medium with 10% CCK-8 fluid was used to replace the eluates, and the cells were incubated for 2 h. Optical absorbance (OD) of the medium was then measured at 450 nm.

### Light transmission, color change and stability

Resin disks were prepared using molds with a diameter of 9 mm and thickness of 1, 2, 3, and 4 mm (*n* = 6). The light transmission through the resin disks was measured three times for each sample, and the light source used was Elipar S10, 3 M, USA. After blank measurement for calibration, the transmitted light through each disk as the luminous flux in mW/cm^2^ was received by the radiometer detector (TA632A, TASI, China). The rate of percentage transmittance was calculated.

Resin disks were prepared using molds with a diameter of 9 mm and thickness of 2 mm (*n* = 6). Color change induced by incorporating DMAHDM and color stability after water and coffee storage were analyzed using a spectrophotometer (SpectraScan PR-670, Photo Research, USA). The measurement was performed under the D65 standard light in a light box (CAC-600-1200L, JiaBiao, China), and the specimens were placed on a gray standard. The total color variation was calculated according to the following formula:

$$\Delta E = \sqrt {\left( {\Delta L^2} \right) + \left( {\Delta a^2} \right) + \left( {\Delta b^2} \right)}$$

The color change measurement was conducted after the specimens were prepared. The color of these samples was tested and marked as T1, and the total color variation, which was compared with the Control group was determined.

The specimens were immersed in DDW at 37 °C for 4 weeks. The DDW was refreshed every week [23]. After 4 weeks, the color of the samples was read. The total color variation of each group, which was compared with the value itself in T1, was calculated.

For the coffee aging, the samples of each group were immersed in coffee (10.0 wt/v%) (Nescafé; Nestlé, Switzerland) for 72 h to simulate the consumption of coffee for 3 months [24]. Then the specimens were rinsed with DDW for 5 min and air dried. Next, the color of the samples was read. The total color variation of each group, which was compared with the value itself in T1, was calculated.

### Bacterial culture and biofilm formation on specimens

Resin disks with diameter of 9 mm and thickness of 1 mm were made. The use of the dental plaque microcosm biofilms with human saliva as inoculum was approved by the Ethical Committee of the School of Stomatology, Fujian Medical University (202145). Ten healthy volunteers were chosen as donors who had natural dentition, were without periodontal disease and active caries, and had not taken any antibiotics in the previous 3 months. An identical volume of saliva from each donor was pooled together and diluted two-fold with sterile 50% glycerol, which was stored at −80 °C [30].

A McBain artificial saliva medium (1.5 mL) was added to each well of 24-well plates with a composite disk (*n* = 6). The medium consisting of 2.5 g/L mucin, 2.0 g/L Bacto peptone, 2.0 g/L Trypticase Peptone, 1.0 g/L yeast extract, 0.35 g/L NaCl, 0.2 g/L KCl, 0.2 g/L CaCl_2_, 0.001 g/L hemin, and 0.0002 g/l vitamin K1, with 0.2% sucrose and 50 mmol/l PIPES at pH 7.0 [31]. The saliva-glycerol stock was added with 1:50 final dilution. To better replicate conditions existing in supragingival plaque, the saliva-derived biofilms were incubated in an anaerobic environment [32]. After 24 h, the composite disks with biofilms were transferred to new 24-well plates with fresh medium, and incubated for another 24 h [33]. This totaled a culture time of 48 h, which was sufficient to form microcosm biofilms on the resins [30].

### MTT metabolic assay of biofilms

Composite disks were inoculated with saliva-derived bacteria and cultured for 48 h. The biofilms were washed twice with PBS, placed in 24-well plates (*n* = 6), and inoculated with 1 mL of MTT solution (with 0.5 mg/mL MTT in PBS) for 4 h at 37 °C in 5% CO_2_ [34]. Then the disks were transferred into new 24-well plate, and 1 mL of dimethyl sulfoxide (DMSO) was added to dissolve the formazan crystals. The plates were incubated for 20 min with gentle mixing. Two hundred microliters of the DMSO solution were then transferred into 96-well plates, and OD_540nm_ was determined using a microplate reader (MD SpectraMax iD3, Molecular Devices, USA) [34].

### Live/dead bacterial assay

The composite disks with 48 h biofilms were washed three times. Three specimens per group were stained with BacLight live/dead bacterial viability kit (Molecular Probes, Eugene, USA) [35]. Each specimen was photographed in five randomly-selected fields of view. Live bacteria were stained with SYTO 9 to produce a green fluorescence. The compromised bacteria were stained with propidium iodide (PI) to produce a red fluorescence. An inverted epifluorescence microscope (IX71, Olympus, Japan) was used to image the biofilms.

### Flow-cytometric assay for biofilms on resins

The biofilms were washed three times with PBS. Then the biofilm of each group was collected in 1 mL PBS by scrape and sonication. PI and SYTO 9 were then added to each of the biofilm collection and incubated in dark and at 37 °C for 15 min. The biofilms were analyzed with BD Accuri C6 plus system (BD Biosciences). Green and red fluorescence were detected at 500 nm (FL1 channel) and 610 nm (SSC-A channel).

### Statistical analysis

Kolmogorov–Smirnov test was used to check normal distribution of all the data. One-way analyses of variance with Tukey’s honestly significant difference were performed for comparison. The statistical software SPSS 22.0 (SPSS Inc., USA) was used. *p* < 0.05 was considered to be significant.

## Results

**Fig. 2:**
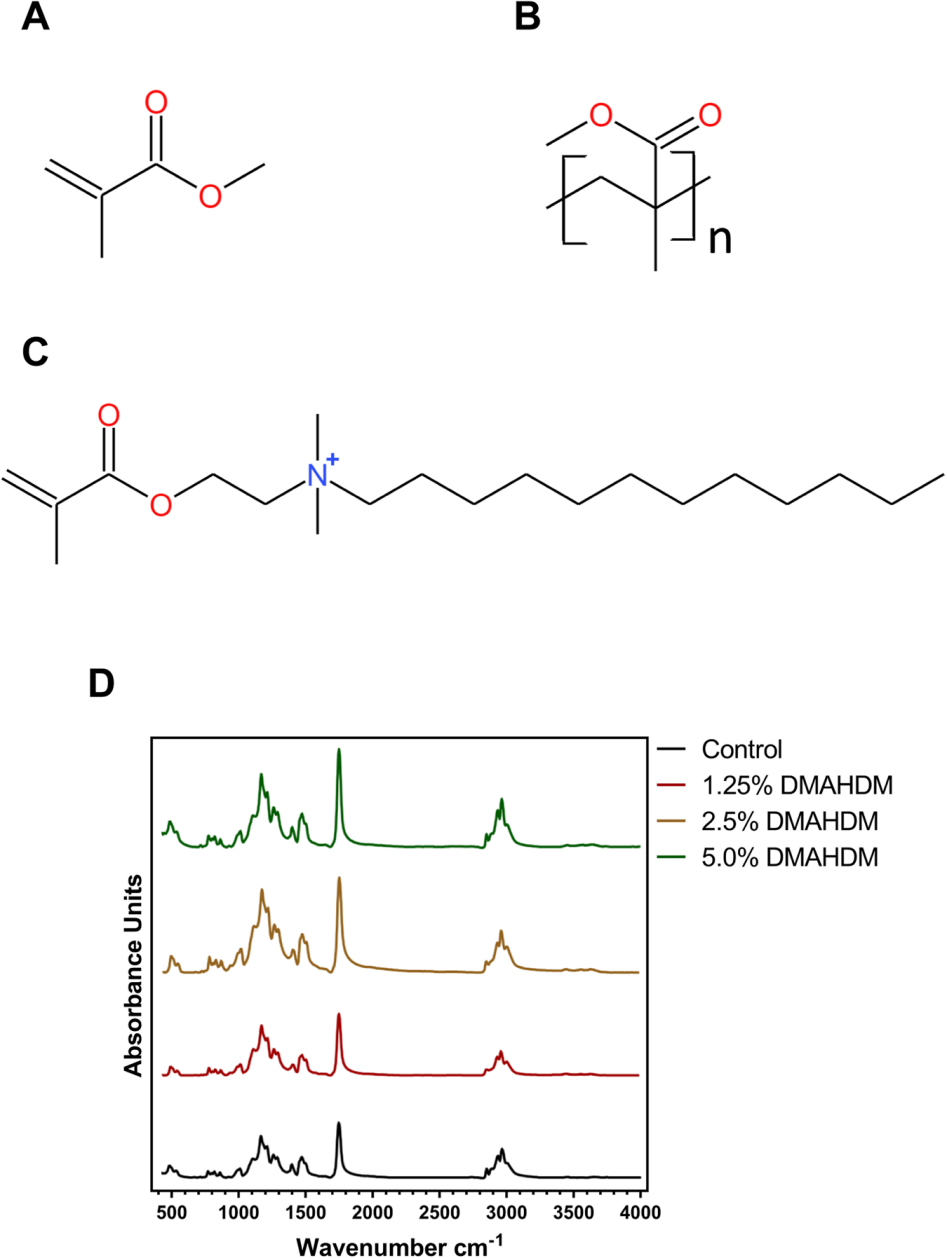
Chemical components of the resins. **A** Chemical structure formula of MMA monomer; **B** polymethyl methacrylate (PMMA); **C** dimethylaminohexadecyl methacrylate (DMAHDM); **D** FTIR spectra of the self-cured resins

Figure 2 shows the chemical components of the antibacterial resin. MMA and DMAHDM both have the group of methacrylate. FTIR spectra of the self-cured resins showed that no obvious other new peak was present in the modified resin when compared to Control. It indicated the chemical stability of the modified resin. The peak intensity of the antibacterial resins was getting higher as the DMAHDM concentration increased.

**Fig. 3:**
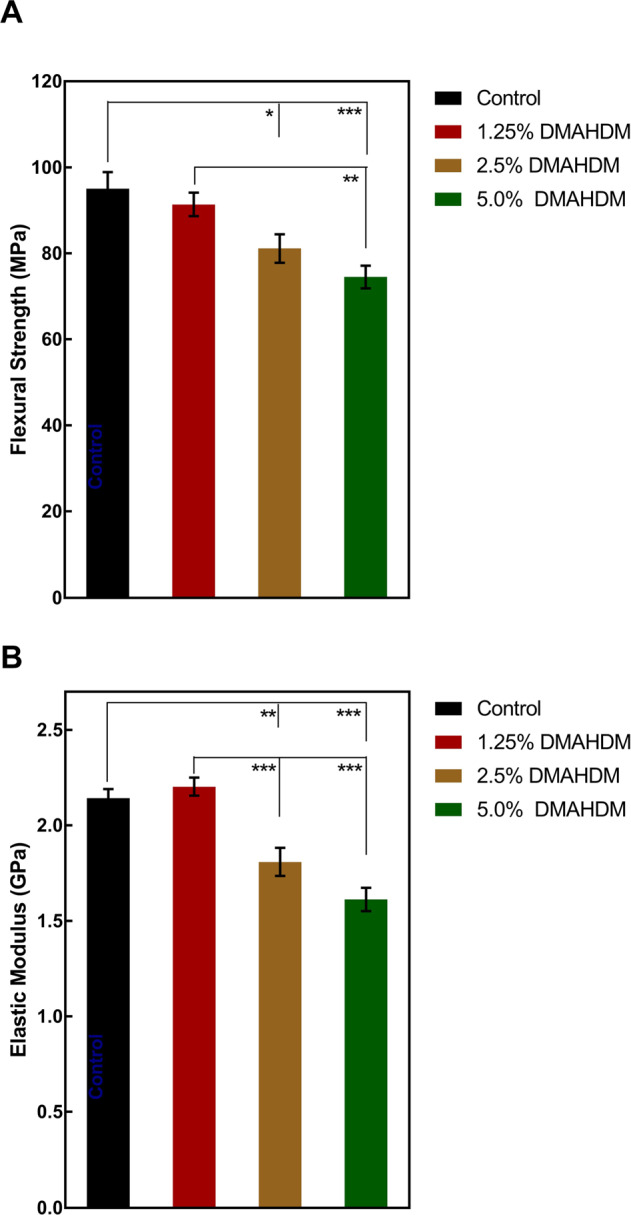
Mechanical properties of composites. **A** Flexural strength, and (**B**) elastic modulus (mean ± SD; *n* = 6). (*p < 0.05. **p < 0.01. ***p < 0.001)

The mechanical properties of the antibacterial resins are plotted in Fig. 3 (mean ± standard deviation (SD); *n* = 6). Compared to Control, the incorporation of 1.25% DMAHDM had no adverse effect on flexural strength and elastic modulus (*p* > 0.05). However, the resins in group 2.5% and 5% showed decreased mechanical properties (*p* < 0.01). Taking flexural strength as an example, the value of Control, 1.25% DMAHDM, 2.5% DMAHDM, and 5% DMAHDM were 95.04 ± 11.02 MPa, 91.38 ± 7.74 MPa, 81.15 ± 9.29 MPa, and 74.52 ± 7.43 MPa, respectively.

**Fig. 4:**
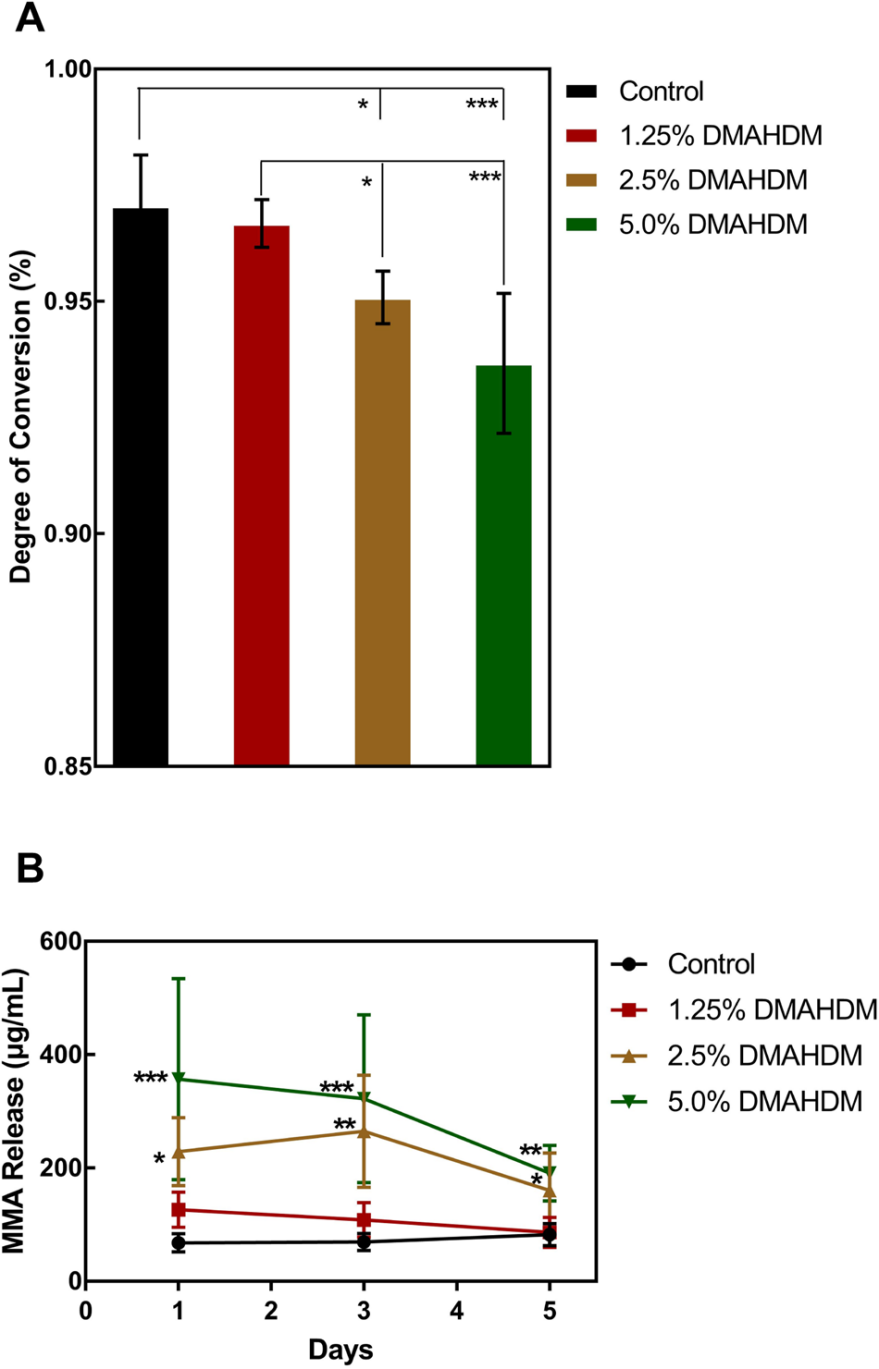
Degree of conversion (DC) and unpolymerized monomers releasing. **A** DC, and (**B**) MMA release. (mean ± SD; *n* = 6. *p < 0.05. **p < 0.01. ***p < 0.001)

As shown in Fig. 4A, DC values of Control and 1.25% DMAHDM groups are 97.06 ± 1.00% and 96.68 ± 0.47%, respectively. DC value in 5.0% DMAHDM is the lowest (*p* < 0.05), 93.68 ± 1.38%. Future, the value in 2.5% DMAHDM, 95.01 ± 0.51%, is also lower when compared with Control and 1.25% DMAHDM (*p* < 0.05).

Throughout 5 days of elution, the concentration of the MMA in the eluates progressively reduced. At each time point, the concentration of MMA was positively related with the mass fraction of DMAHDM. For example, 5.0% DMAHDM held the highest value (*p* < 0.001), 322.01 ± 135.36 μg/mL at 3 days, and the concentration of 2.5% DMAHDM, 1.25% DMAHDM, and Control was 264.93 ± 90.70 μg/mL, 108.15 ± 28.05 μg/mL, and 69.02 ± 14.05 μg/mL, respectively. In addition, throughout the 5 days, no statistical difference was observed between 1.25% DMAHDM and the Control group (*p* > 0.05).

**Fig. 5:**
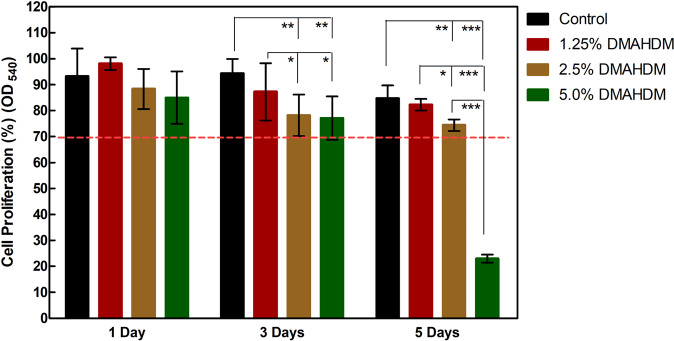
Cytotoxicity against human gingival fibroblasts (HGFs)

The cytotoxicity of the eluates of resins is shown in Fig. 5. From the third day, the monomers released from 2.5% to 5% DMAHDM obviously inhibited cell proliferation when compared with the Control group (*p* < 0.05). Moreover, according to the ISO criteria, when cells’ survival rates were over 70%, this represented low cytotoxicity [36]. The cells’ survival rates in the Control, 1.25%, and 2.5% DMAHDM groups stayed higher than 70% throughout the 5 days. For example, at 5 days, the cell proliferation in the Control, 1.25%, and 2.5% DMAHDM groups were (84.60 ± 4.60)%, (82.24 ± 2.05)%, and (74.35 ± 2.06)%, while 5% DMAHDM was (22.93 ± 1.41)%. These results suggest that the former three resins are of low toxicity to HGFs.

**Fig. 6:**
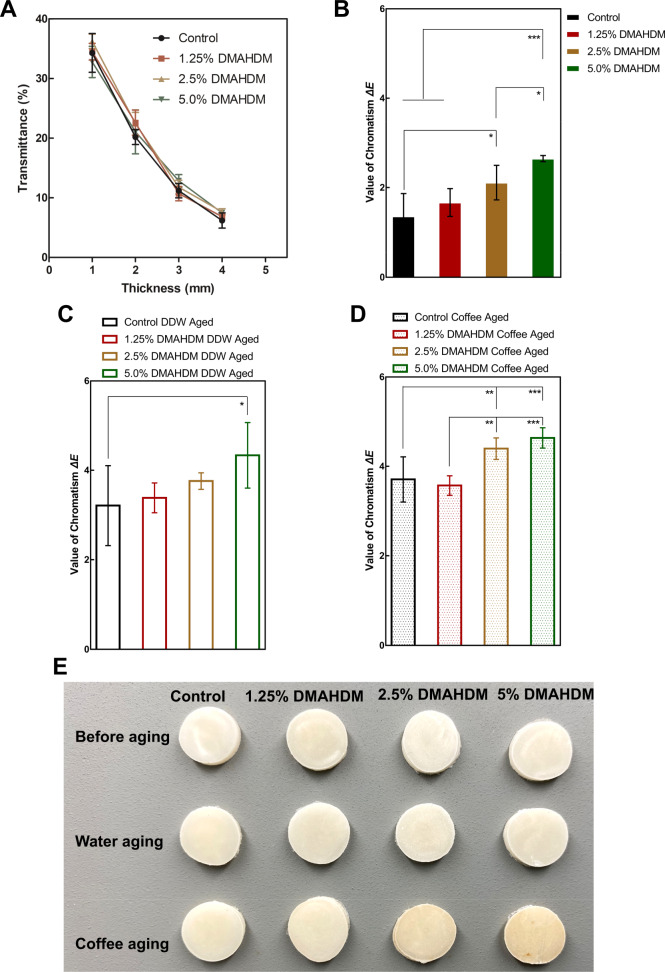
Light transmission, color change and stability (∆*E*) of resins. **A** Transmittance spectrum of the resins; **B** color change of the antibacterial resins compared with Control resin; **C** ∆*E* of resins after deionized water (DDW) aging; **D** ∆*E* of resins after staining by coffee; **E** represented image of the resins before and after aging. (mean ± SD; *n* = 6. *p < 0.05. **p < 0.01. ***p < 0.001)

The percentage of transmitted light at each sample in different thicknesses can be observed in Fig. 6A. It was demonstrated that adding DMAHDM into the PMMA resins had no influence on the light transmission properties. According to Fig. 6B, adding DMAHDM would induce color change of the resin, and the ∆*E* of 2.5% and 5% DMAHDM was obviously increased (*p* < 0.05). However, 1.25% DMAHDM showed no statistically significant color change when compared with Control (*p* > 0.05). The ∆*E* values after DDW storage were plotted in Fig. 6C, and resins in the 5% DMAHDM group held the greatest ∆*E* value (*p* < 0.05), indicating the most obvious color change among the four groups. Figure 6D represents the condition of color stability after coffee aging. The ∆*E* of 2.5% (4.40 ± 0.22) and 5% DMAHDM (4.64 ± 0.20) were obviously higher than in 1.25% DMAHDM (3.57 ± 0.20) and Control (3.71 ± 0.46) (*p* < 0.01), and the ∆*E* in 1.25% DMAHDM and Control showed no difference (*p* > 0.05). The represented image of the resins before and after aging are shown in Fig. 6E. The color change condition can be observed in accordance with the quantity analysis in the images. Therefore, 1.25% DMAHDM showed similar color change and stability as Control resin. However, higher DMAHDM would hinder these aesthetic properties of the resins.

**Fig. 7:**
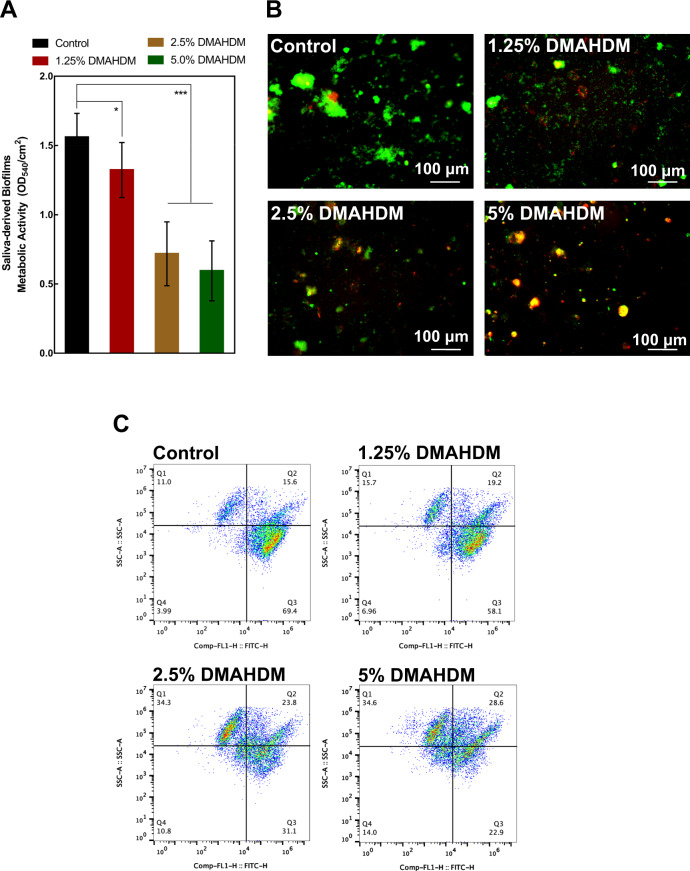
Inhibition effects of resins against saliva-derived biofilms (mean ± SD; *n* = 6). **A** Metabolic activity; **B** representative images of live/dead stained saliva-derived biofilms on resins; **C** flow cytometric analysis of saliva-derived biofilms on the resins. The four quadrants represent different viability stages of bacteria: Q1: dead bacteria; Q2: injured bacteria; Q3: live bacteria; Q4: weakly fluorescent debris. Live bacteria were stained green, and dead bacteria were stained red. (mean ± SD; *n* = 6. *p < 0.05. **p < 0.01. ***p < 0.001)

The metabolic activity levels of saliva-derived biofilms on the resins are plotted in Fig. 7A (mean ± SD; *n* = 6). The resins containing DMAHDM greatly reduced the metabolic activity of biofilms (*p* < 0.05), and the inhibition effects were positively related to the concentration of DMAHDM. When compared with control resin, the metabolism level of saliva-derived biofilms on the 1.25%, 2.5%, and 5% DMAHDM resins were reduced by 20%, 54%, and 62%, respectively.

Figure 7B shows representative live/dead images of the saliva-derived biofilms on the resins. The Control resin was covered almost completely by live bacteria. In contrast, less biofilm was observed on 1.25% DMAHDM resins, and more dead bacteria were present. This trend was more obvious on the 2.5% and 5% DMAHDM resins.

Figure 7C plots the flow cytometric analysis of saliva-derived biofilms on the resins. There was a gradual shift of bacteria population from viable to dead and injured cells with an increase in mass ratio of DMAHDM. For example, the percentage of dead cells increased gradually from 11.0% (Control) to 15.7% (1.25% DMAHDM), 34.3% (2.5% DMAHDM), and 34.6% (5% DMAHDM).

## Discussion

The present study incorporates DMAHDM into the autopolymerizing PMMA resin at low mass fraction. The hypotheses were proven that low concentration of DMAHDM successfully conferred antibacterial effectiveness to the modified resin and had no negative influence on the materials’ mechanical, chemical, and aesthetic properties and biocompatibilities.

The MMA and DMAHDM monomers both have the group of methacrylate. The aliphatic (C=C) double bonds in these monomers have the potential to copolymerize with each other, thus achieving chemical immobilization of antibacterial DMAHDM [21, 22]. The chemical analysis of the modified resins showed that no new chemical element could be detected by FTIR. This demonstrated that the chemical interference of the modified resin was limited. This lay the foundation for maintaining the mechanical and aesthetic properties and biocompatibilities of the resin.

Possible toxic reactions are of major concerns when DMAHDM is incorporated into PMMA resin. Therefore, a cytotoxicity test was performed. All groups but 5% DMAHDM showed cell viability higher than 70% for the eluate of all time points. As stated in ISO 10993, 70% cell viability is the standard for non-cytotoxicity [36]. Thus, lower mass ratio of DMAHDM at 1.25% and 2.5% meets the ISO criteria, and more importantly, no statistical difference was detected between the Control and 1.25% DMAHDM groups. This modified antibacterial resin had the same biocompatibility as the commercial Control. The resin containing 5% DMAHDM exhibited great cytotoxicity which is consistent with previous studies [23]. The low DC and more residual MMA and DMAHDM monomers might be the reason for the unsatisfied biocompatibility of the resin in the 5% DMAHDM group.

The DC of PMMA resin is far from 100%, therefore, acrylic resins are known to contain and release unpolymerized monomers [37], and there is an inverse relationship between DC and the amount of residual monomers [7]. These acrylic monomers are reported to be cytotoxic in vitro [37]. Determination of the MMA leaching is one method to investigate the in vitro cytotoxicity of PMMA resins [7, 37]. Therefore, in the present study, DC and MMA release was measured. It turns out that the 1.25% DMAHDM resin held a similar DC and MMA monomer release level to commercial Control resin. This demonstrates the acceptable chemical properties of the modified resin. However, when the mass ratio increased to 2.5% and 5%, more precipitates were presented in the liquid, indicating the mass ratio exceeded the solubility of DMAHDM in the liquid MMA monomer [23]. Further, the undissolved DMAHDM can not fully react with the MMA monomers; after leaching out of these unpolymerized monomers, pores might be left in the resin. In addition, the higher mass ratio of DMAHDM means a high density of long alkyl chain, and the steric hindrance effect of the long chain might inhibit the copolymerization between the monomers. Therefore, lower DC and higher MMA release can be detected in the 2.5% and 5% DMAHDM groups. And it was observed that the MMA release decreased with 5 days. The reductions might be a result of further polymerization of residual monomers and the decrease in monomers left in the PMMA resin. Degradation of methacrylates by oxidation reactions or hydrolysis of functional groups over time, might also explain the reduction of residual monomers [37]. Moreover, some of the degradation by-products and other leaching compounds, such as additives, impurities, and decomposed products might also lead to the in vitro cytotoxicity of PMMA resins [7, 37]. It should not be neglected that the unpolymerized DMAHDM monomers in the modified resins may also be related to the cytotoxicity. In the future studies, the toxicity of DMAHDM in the modified PMMA resin should be investigated and clarified.

As for the mechanical properties of the modified resin, the incorporation of 1.25% DMAHDM had no adverse effect on flexural strength and elastic modulus. However, 2.5% and 5% DMAHDM showed inferior mechanical properties. This might be related to the difficulty of dissolving DMAHDM in the acrylic resin, result in the formation of clusters of this component and higher porosity [23]. Lower DC also leads to hampered physical strength of the modified resin [7, 26].That said, in the present study, all groups of the acrylic resin, including 5% DMAHDM, achieved flexural strength of more than 70 MPa, higher than the lowest flexural strength criteria of this kind of self-cured material (60 MPa) set by ISO 20795-1 in 2013 [27]. This result is different from the previous study, in which the flexural strength of 5% DMAHDM resin was <60 MPa [23]. This might be owing to the post-polymerization heat treatment help, improving the DC while reducing the residual MMA [7, 26]. As a consequence, the mechanical properties of the treated resin were also enhanced.

Biofilm accumulation on the provisional restorations causes gingival inflammation, denture stomatitis, and secondary caries [2]. Using the biofilm model, which mimics the circumstances in the oral cavity, is a proper way to study antibacterial properties of the modified resin [38]. Previous studies demonstrated that stable microcosm oral biofilms can be produced from human saliva samples [32, 38, 39]. Microcosm biofilms from human saliva are similar to natural plaque [40]. In the present study, the saliva was donated by ten donors and then was then mixed to achieve the diversity and heterogeneity for the biofilm model. This might overcome the drawbacks encountered in studying in vivo biofilms, such as lack of standardization among subject characteristics.

The incorporation of DMAHDM into the PMMA resin successfully rendered the modified resin with anti-saliva-derived biofilms effective. When compared with control resin, the metabolism level of saliva-derived biofilms on the 1.25%, 2.5% and 5% DMAHDM resins was reduced by 20%, 54%, and 62%, respectively. As regards the live/dead staining of the saliva-derived biofilms on the resins, a similar trend could be observed, in that the Control resin was covered almost entirely by live bacteria. In contrast, less biofilm was observed on the DMAHDM-modified resins, and more dead bacteria were present. This trend was more obvious as the concentration of DMAHDM increased. This indicated that the modified resins can effectively reduce the oral biofilm formation and the metabolism activity. Therefore, the DMAHDM-improved resins have the potential to inhibit the occurrence of gingival inflammation, denture stomatitis, and secondary caries, which are caused by biofilm accumulation on the provisional restorations. The positively charged quaternary amine N^+^ of DMAHDM can interact with the negatively charged cell membrane of bacteria, causing destruction of membrane and leading to an outburst of cytoplasm [41]. In addition, the long chains of DMAHDM might be able to insert into bacterial membranes to disrupt bacteria [41]. To gain further insight and confirm the bacteria-killing mechanism of DMAHDM, flow cytometric investigation and live/dead staining of biofilms grown on the resins were performed. The fluorescent dye PI enters only permeabilized cells, binds DNA, and fluoresces [42]. In dead and injured cells, PI enters the cell through damaged membrane and binds DNA, which makes it fluoresce [42]. In intact viable cells, PI remains in the medium and does not fluoresce [42]. The results of the present study evidence that adding DMAHDM in PMMA resin can lead to an increase of compromised bacteria in the saliva-derived biofilms, and the percentage of dead and injured cells increased gradually with the higher mass ratio of DMAHDM in the modified resins. What is more, previous studies demonstrated that by copolymerizing with the resin, the DMAHDM is immobilized in resin and does not leach out over time, thus providing a durable antibacterial capability and mechanical properties [43, 44].

In aesthetically critical areas, the provisional restoration must not only provide an initial shade match but also maintain an aesthetic appearance over the period of service. Discoloration of provisional materials for fixed prosthodontics may result in patient dissatisfaction and additional expense for replacement. Hence, color stainability may be a significant criterion in the selection of a particular provisional material for use in an aesthetically critical area [24]. The present study demonstrated that adding a high mass ratio of DMAHDM up to 5% would not induce immediate color change of the modified resin. However, high proportion of DMAHDM would negatively affect the color stability of the resin. Fortunately, when the mass ratio was decreased to 1.25%, the ∆*E* value decreased to a similar level to the control resin after water and coffee aging. It is reported that the degree of color change is affected by a number of factors such as DC, water sorption, chemical reaction and surface roughness of the restoration, as well as diet and oral hygiene of the patients [45]. In the present study, a possible reason for this could be that the high ratio of DMAHDM negatively influenced the physicochemical properties of the modified resins.

Based on these results, the PMMA resins containing lower mass ratio of DMAHDM successfully inhibited saliva-derived biofilms while maintaining the mechanical, chemical and aesthetic properties. However, it cannot be neglected that the present study was performed in in vitro condition, which is unable to perfectly mimic the real condition in the oral cavity. In addition, further study was needed to explore the mechanical and chemical properties, color stability, and antibacterial activity of the modified resins after long-term aging, because some of the provisional restorations were subjected to prolonged usage in clinic.

## Conclusions

This study showed that the PMMA resins containing lower ratio of DMAHDM inhibited saliva-derived biofilms and maintained the mechanical, chemical, and esthetic properties. DMAHDM effectively disrupted the saliva-derived biofilm formation, and the mechanism of DMAHDM disturbing the integrity of the bacterial cell wall was confirmed in the present study. Although more clinically relevant studies are still needed, this novel antibacterial provisional restoration material is promising to help inhibit bacterial invasions and optimize the prognosis of provisional PMMA restorations and the following dental treatments.
